# A Method for Measuring the Operating Force of Interventional Robots via Integration of Compliant Mechanisms and Sensors

**DOI:** 10.3390/biomimetics11040229

**Published:** 2026-03-31

**Authors:** Baozhen Ren, Hui Li, Yongliang Cao, Chang Wang, Yan Zhao, Jianhua Zhang

**Affiliations:** 1 School of Mechanical Engineering, Hebei University of Technology, Tianjin 300401, China; 202211201005@stu.hebut.edu.cn; 2 School of Mechanical Engineering, University of Science and Technology Beijing, Beijing 100083, China; d202310347@xs.ustb.edu.cn (Y.C.); wangchang@ustb.edu.cn (C.W.); yanzhao@ustb.edu.cn (Y.Z.); jhzhang@hebut.edu.cn (J.Z.)

**Keywords:** interventional robot, operating force detection, compliant mechanism, master controller

## Abstract

Interventional robots play a crucial role in surgical procedures, where accurate force measurement is essential for enhancing safety. Compliant mechanisms utilize material deformation to achieve millinewton-scale force output and millimeter-level displacement with high repeatability. Motivated by this, we propose a method for measuring the catheter force by integrating a compliant mechanism and a sensor. First, we designed an operating force detection module. It comprises a double-parallelogram structure with four elastic units, a catheter drive module, and a sensor. The sensor connects the compliant mechanism to the base. Second, stiffness and gravity compensation models were established and validated experimentally. Finally, we constructed an experimental platform to evaluate the force measurement accuracy, drive accuracy, and real-time detection capability. Experimental results demonstrate that the proposed method achieves a maximum detection error of 0.1482 N, an average error of 0.0096 N, a resolution of 0.01 N, and an average axial delivery error of 0.8287 mm. Additionally, a master–slave control framework was developed, along with a master controller that manipulates the slave robot to deliver the catheter within a vascular phantom, while simultaneously displaying real-time force information via the human–computer interaction interface.

## 1. Introduction

Interventional surgery, as a minimally invasive treatment, has been widely utilized in cardiovascular, neurological, and other medical fields in recent years. Robot-assisted interventional surgery can enhance procedural accuracy and stability while reducing physicians’ exposure time to radiation [1,2]. During interventional procedures, accurately detecting the operating force is essential. It is not only the core prerequisite for achieving closed-loop control of master–slave operations and ensuring accurate replication of surgical actions [3,4], but also the key basis for doctors to perceive the intravascular environment and avoid excessive operation that may cause tissue damage [5,6].

Several FDA-approved interventional robotic systems, including Sensei X [7] and Magellan [8] (developed by Hansen Medical, Mountain View, CA, USA), the Niobe robotic magnetic navigation system [9] (Stereotaxis, St. Louis, MO, USA), and CorPath GRX [10], have already been translated into clinical practice. However, these systems do not incorporate force-sensing capabilities for surgical instruments, and further improvements are required to enhance procedural safety. Extensive research has been conducted on detecting operating forces in interventional robots [11,12,13,14,15,16]. Yan et al. [17] designed a gripper that mimics a surgeon’s fingers and developed an orthogonal force-coupling model to describe the relationship between clamping and insertion forces. Lv et al. [18] proposed an intravascular interventional robot with a soft clamp, which takes motion and clamp deformation as input and outputs insertion force and torque through LSTM. Bao et al. [3] proposed a reciprocating dragging robot, which uses a pull pressure sensor to directly measure the interaction force between the clamping and driving modules. Woo et al. [19] proposed a reciprocating pull–push interventional robot that employs a six-axis force/torque sensor to enable direct measurement of catheter manipulation forces. Ishihara et al. [20] developed a vascular interventional robotic system incorporating visual force feedback, in which an L-shaped gripper mounted on a linear guide rail is used for catheter advancement. Similar to the reciprocating pull–push approach, this configuration is inherently influenced by frictional forces within the guide rail transmission. Yu et al. [21] performed static and dynamic compensation for the inertial force and instrument friction of the reciprocating dragging robot, and used non-parametric regression to compensate for vascular contact force and blood viscosity. Wang et al. [22] proposed an enhanced force feedback strategy based on distal force prediction, combining the time convolution network (TCN) prediction with the spring-damper model to map the force state, thereby improving the accuracy of force perception. Ren et al. [23,24] proposed a multi-point bending contact force detection method for the catheter of rotary clamp delivery, which was verified to be effective by ANSYS Workbench 2020 R2 simulation and experiments. Zhang et al. [25] simulated the shape of the guidewire through discrete parameterization and Bézier curves and established a model to estimate the contact force. Nguyen et al. [26] developed a miniature soft robotic catheter that integrates a hydraulically actuated artificial-muscle-driven, omnidirectionally expandable flexible manipulator, a lantern-shaped variable-stiffness stabilization mechanism, and a high-sensitivity soft force sensor, thereby enabling accurate steerable navigation, stable intraoperative positioning, and real-time monitoring of tool–tissue interaction forces during vascular interventions. Nevertheless, the system exhibits minor force-sensor drift and hysteresis in the variable-stiffness mechanism. Dagnino et al. [27] proposed an MRI-compatible, pneumatically actuated vascular interventional robot and validated it through four in vivo animal studies; however, the platform lacks a dedicated mechanism for operative force sensing. In addition, magnetically driven interventional robots have been a hot topic in recent years [28,29]. Using mathematical modeling methods, magnetically driven robots can obtain the tip contact force [30,31]. Gunduz et al. [32] proposed a miniaturized magnetically actuated guidewire robot, offering new possibilities for minimally invasive endovascular therapy and showing potential to become a key enabling technology for next-generation image-guided interventions; however, it still faces significant challenges in terms of technical maturity, clinical validation, and translational deployment. Sa et al. [33] introduced a separable and reconfigurable magnetic robot that realizes catheter steering via the interaction between an external magnetic field (EMF) and embedded permanent magnets, and enables helical decoupling of the untethered magnetic robot from the delivery catheter, as well as thrombus tunneling, through a rotating EMF; however, no method for operative force sensing was reported. In short, strong magnetic fields may be attenuated or distorted when penetrating human tissue, and the positioning accuracy in tissues such as blood vessels needs to be improved [34]. Mehdi et al. [35] proposed an integrated actuation and sensing system based on FBG optical fibers, using the FBG fibers as tendons combined with pneumatic artificial muscles to achieve distal catheter bending control. They also converted FBG strain measurements into tension and built a data-driven contact force detection model based on an LSTM network. However, the system’s performance was only verified at low frequencies (≤0.8 Hz), and stability in high-frequency scenarios needs improvement. Additionally, contact detection relied solely on force signals without integrating multi-dimensional information, resulting in limited anti-interference capability. Hassen et al. [36] proposed a three-degree-of-freedom soft catheter force sensor based on three FBG optical fibers, but its axial force accuracy was affected by the viscoelasticity of soft materials and assembly errors, and it lacked a temperature compensation mechanism. Arefinia et al. [37] proposed a CNN-LSTM-based multimodal sensor-less force estimation method, which was limited to two-dimensional force estimation, did not account for blood fluid resistance, was only suitable for unidirectional catheters, and relied on static camera imaging. In summary, existing operating force detection methods have problems such as lag and insufficient accuracy, and the detection accuracy and sensitivity need to be further improved.

Compliant mechanisms use material deformation to transmit or convert motion and force. Reciprocating drag-type operating force detection scheme involves mounting the clamping module on the slide of the guide rail. The clamping module is connected to the fixed base through a sensor, and the force detected by the sensor does not include the friction between the slide and the guide rail, which is difficult to analyze through theoretical models. Compared with the reciprocating drag-type operating force detection scheme, using a compliant mechanism to connect the clamping module and the base avoids issues of clearance, friction, wear, or lubrication [38,39]. Current research on compliant mechanisms for force sensing has established a solid technical foundation. However, studies addressing surgical operation force detection using compliant mechanisms remain limited. Most existing studies primarily focus on force output or micromanipulation. Xu et al. [40] proposed a compliant constant force mechanism suitable for small target operation, which achieves sub-Newton force output and millimeter-level travel. Meanwhile, the output characteristics of the mechanism are less affected by the driving speed and acceleration, and have good repeatability. Inspired by this, researchers employed a series of flexible actuators to achieve precise positional output of a micro-motion platform [41]. Gao et al. [42] applied a compliant mechanism to the puncture force detection system. They proposed a compact two-degree-of-freedom (2-DOF) cross-scale piezoelectric robotic manipulator. The system enables adjustable puncture force. In our previous research, we proposed a rotary clamping delivery interventional robot. This approach offers higher clamping force and greater efficiency than the reciprocating dragging delivery scheme. We also proposed a method for detecting multi-point bending forces in the catheter during rotary clamping-based delivery. However, the method’s sensitivity to subtle force variations remains insufficient. Therefore, we have further refined the force detection scheme based on this approach. In this paper, we proposed a method for detecting the operation force of interventional instruments by connecting a double parallelogram mechanism in series with a force sensor.

In this paper, our main contributions are as follows: This paper proposed a compliant mechanism-sensor integration method for detecting micro-operational forces. By harnessing the mechanism’s deformation to transmit force coupled with high-precision sensing, it overcomes the traditional limitations of poor signal-to-noise ratio and interference in 10 mN force detection, achieving precise capture of subtle contact forces in interventional procedures. The proposed design employs a frictionless compliant mechanism. This mechanism eliminates frictional interference through a transmission method integrating flexible hinges with suspended guidance. By contrast, existing force measurement solutions depend on ball screws and sliding guides. Our design structurally avoids friction, enhancing the precision and stability of interventional force detection. We established a mapping model that describes the relationship between mechanism compliance and operational force. First, the analytical relationship between deformation displacement and actual force was derived by integrating theoretical modeling with experimental calibration. This step provided crucial theoretical support for micro-force detection. Consequently, the model overcomes the limitations of traditional flexible mechanisms, which often rely on ambiguous and empirically calibrated force-deformation relationships. As a result, our detection system maintains consistent accuracy across diverse interventional scenarios, significantly enhancing its universality.

The content structure of this paper is organized as follows: Firstly, we comprehensively explain the structural design of the force sensing module, the principle of force detection, the theoretical model analysis, and the structural design optimization method. Secondly, the effectiveness of the proposed method is examined through experiments, which include the verification of the theoretical model, experimental testing of the performance of the force detection module, and in-depth analysis of the experimental results. Finally, we compare the results with existing research and conclude this article.

## 2. Design and Analysis of Force-Sensing Schemes

### 2.1. Structural Design of the Drive Module

**Figure 1:**
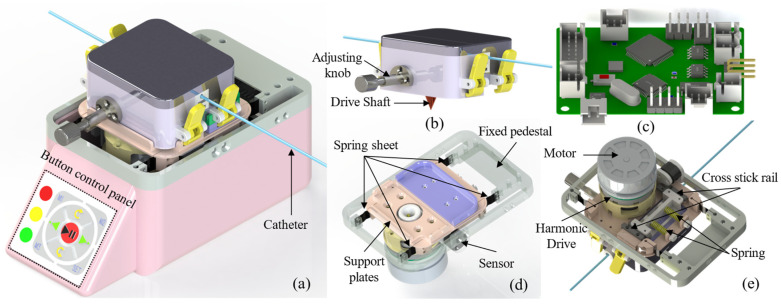
Design details of the catheter delivery module and the force detection scheme (**a**) Three-dimensional structure of the catheter delivery module. (**b**) Three-dimensional view of the clamping module. (**c**) A printed circuit board (PCB) inside the module is used to acquire force sensor signals and output key I/O control parameters. (**d**) Schematic of the force detection scheme: the transmission module is suspended and fixed to the base via four compliant units, and the sensor is mounted on one side of the module. (**e**) The internal schematic of the transmission module primarily includes the drive motor, harmonic reducer, and adjustable clamping mechanism.

This paper proposes an improved design for the catheter operation module in a rotary clamping delivery system. The structure of the catheter delivery module is shown in Figure 1a, consisting primarily of a clamping module (Figure 1b) and a transmission module (Figure 1d). The clamping module is composed of active and passive clamping units. The active clamping unit is connected to the transmission module via a quick-release mechanism, while the passive clamping unit is guided by a cross-stick guide rail, as illustrated in Figure 1e. It remains clamped due to the spring preload. The transmission module consists of a motor (EC45 flat, rated speed 4560 rpm, rated torque 167 mNm, rated voltage 24 V, Maxon, Sachseln, Switzerland) and a harmonic reducer (SHDmini-08-50, Dongguan Xintuo Intelligent Machinery Technology Co., Ltd., Dongguan, China). The clamping and transmission modules are mounted on a support plate, as shown in Figure 1d. The support plate is suspended and secured to the base by four metal spring sheets. A tension and compression sensor (SBT674, range: 2 kg, Nonlinearity: 0.1%F.S., Sensitivity: 0.5 mV/V, hysteresis: 0.05%F.S., Guangzhou Sparto Electronic Technology Co., Ltd., Guangzhou, China) is mounted between the support plate and the base. The reaction force exerted by the catheter on the drive mechanism induces strain in both the pressure sensor and the thin metal sheets. The strain in the tensile sensor leads to an electrical signal variation, enabling direct force measurement, whereas the force acting on the metal sheets requires further analysis. A custom PCB for sensor signal acquisition was designed to ensure compactness, as shown in Figure 1c.

### 2.2. Principles and Analysis of Operating Force Detection

**Figure 2:**
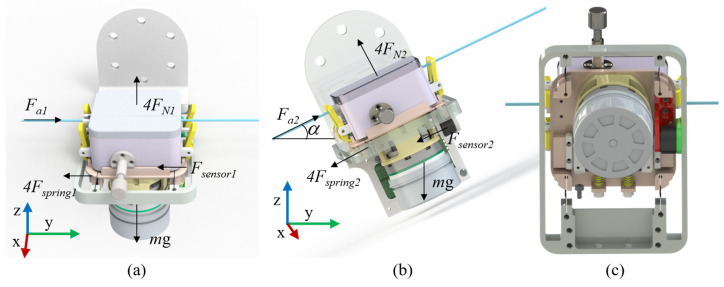
Force Analysis Diagram of the Delivery Module. (**a**) Horizontal installation. (**b**) Tilt installation. (**c**) Schematic Diagram of Sensor Installation.

We utilize a combination of spring sheets and sensors to measure the axial operating force of the catheter. The underlying principle is as follows: when the catheter encounters axial resistance, the force is transmitted to the spring sheets through the clamping module and subsequently to the tension–compression sensor. The four spring sheets supporting the clamping module absorb some axial operating force during this process. In contrast to guide rail–slider mechanisms, the frictional forces between sliders and rails are difficult to quantify accurately through numerical simulation or experimental methods. The proposed method enables establishing a stiffness model for the spring sheets and quantifying the relationship between their deformation and the applied force. The analytical process is as follows. According to the actual working conditions, the catheter delivery module is divided into two states for force analysis. One is when the delivery module is in a horizontal state. In this state, the direction of gravity is perpendicular to the axial force, so it does not affect the axial force. Therefore, the force analysis schematic is shown in Figure 2a. The other state is when the delivery module is inclined. In this case, as the inclination angle changes, gravity generates a component, and the force analysis schematic is shown in Figure 2b.

**Figure 3:**
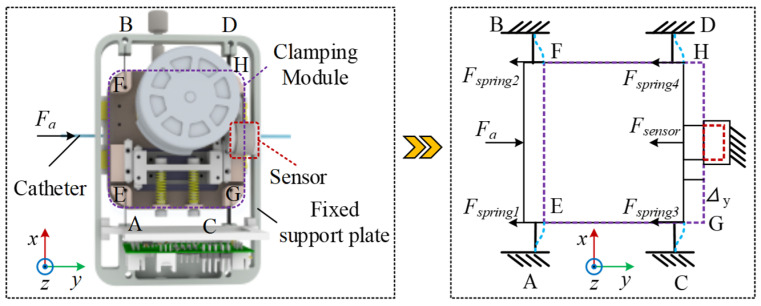
Simplified mechanical model of gripping delivery structure.

For force analysis, the simplification method for the 3D model is as follows: We assume that the entire clamping module is a rigid body, as shown in Figure 3. In the structural design, the clamping module was connected to the sensor through a planar interface to ensure stable and well-defined load transfer. Moreover, reinforcing ribs were incorporated at stress concentration regions to improve the structural integrity of the connecting components, as shown in Figure 2c. This design enables external loads (including gravity and catheter delivery forces) to be transmitted uniformly to the spring element along the prescribed force direction, without introducing noticeable torsional effects. Accordingly, the influence of torsion was neglected in the modeling assumptions. The four points on the fixed plate constraining one end of the spring plate are A, B, C, and D, while the four points connecting the other end of the clamping module to the spring plate are E, F, G, and H, with both ends being fixed-end constraints. When the clamping module is subjected to axial resistance, the displacement of the clamping module is shown by the purple dashed line in the figure, the deformation of the sensor is shown by the red dashed line in the figure, and the deformation of the spring plate is shown by the blue dashed line in the figure. When the clamping module is horizontal, the force equilibrium equation is

(1) $$F_{a1}=4\times F_{spring\;1}+F_{sensor\;1}$$

When the clamping module is tilted, the force balance equation is

(2) $$F_{a2}=4\times F_{spring2}+F_{sensor2}+mg\sin\alpha$$

**Figure 4:**
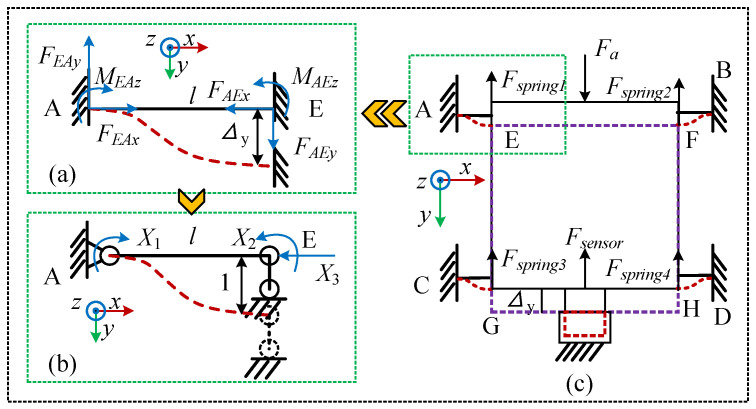
Simplified model and force analysis of a single spring sheet. (**a**) Schematic of a single spring sheet under deformation. (**b**) The force diagram of the spring sheet was simplified using the force method. (**c**) Simplified Overall Free-Body Diagram of the Module.

Take a single spring sheet for analysis, as shown in Figure 4. Since both ends are constrained as fixed supports, its deformation under a displacement load is shown in Figure 4a. This configuration represents a statically indeterminate structure. The force method is applied, with boundary conditions set by assuming zero rotation at the fixed hinge A, zero rotation at the sliding hinge E, and zero horizontal displacement, as illustrated in Figure 4b. The following system of equations is then obtained:

(3) $$\left\{\begin{array}{c} \Delta_{1}=\delta_{11}X_{1}+\delta_{12}X_{2}+\delta_{13}X_{3}+\Delta_{1c}=0\\ \Delta_{2}=\delta_{21}X_{1}+\delta_{22}X_{2}+\delta_{23}X_{3}+\Delta_{2c}=0\\ \Delta_{3}=\delta_{31}X_{1}+\delta_{32}X_{2}+\delta_{33}X_{3}+\Delta_{3c}=0 \end{array}\right.$$

wherein, *X*_1_, *X*_2_, and *X*_3_ represent the removal of redundant constraints, respectively. *δ*_11_, *δ*_12_, and *δ*_13_ represent the displacements along the *X*_1_ direction when *X*_1_, *X*_2_, and *X*_3_ act as unit displacements, respectively; *δ*_21_, *δ*_22_, and *δ*_23_ represent the displacements along the *X*_2_ direction when *X*_1_, *X*_2_, and *X*_3_ act as unit displacements, respectively; *δ*_31_, *δ*_32_, and *δ*_33_ represent the displacements along the *X*_3_ direction when *X*_1_, *X*_2_, and *X*_3_ act as unit displacements, respectively; the generalized displacement parameters Δ_1*c*_ to Δ_3*c*_ represent the rotation about the *X*_1_ axis, the rotation about the *X*_2_ axis, and the translation along the *X*_3_ axis of the elastic unit under unit displacement, respectively. Δ_1_, Δ_2_, and Δ_3_ represent the total displacements in the *X*_1_, *X*_2_, and *X*_3_ directions, respectively.

By applying graphical multiplication method to solve for the coefficients and constant terms in the above system of equations, the following results are obtained:

(4) $$\left\{\begin{array}{l} \delta_{11}=\sum \int \bar{M_{1}}\times \bar{M_{1}}/EIds=l/3EI\\ \delta_{12}=\sum \int \bar{M_{1}}\times \bar{M_{2}}/EIds=l/6EI\\ \delta_{22}=\sum \int \bar{M_{2}}\times \bar{M_{2}}ds=l/3EI\\ \delta_{13}=\delta_{23}=\delta_{33}=0\\ \Delta_{1c}=-\Delta_{2c}=1/l\\ \Delta_{3c}=0 \end{array}\right.$$

wherein, *E* represents the elastic modulus of the spring sheet, *I* represents the moment of inertia of the spring plate, and *l* represents the length of the spring sheet. ${\bar{M}}_{1}$, ${\bar{M}}_{2}$ represent the bending moment diagrams when *X*_1_ and *X*_2_ act individually.

By substituting Equation (4) into Equation (3), the resulting expression is obtained as follows:

(5) $$X_{1}=-6EI/l^{2},\;X_{2}=6EI/l^{2},\;X_{3}=0$$

Subsequently, the shear force in the y-direction of the spring is determined by solving the equilibrium equations.

(6) $$F_{AE}=12EI/l^{3}$$

Therefore, the relationship between sensor strain and delivery resistance is established as follows:

(7) $$F_{a}=(4\times 12EI/l^{3}+k_{sensor})\Delta_{y}$$

Here, $k_{sensor}$ is the stiffness of the sensor $\Delta_{y}$ represents the deformation of the sensor under applied force, $F_{a}$ indicates the axial resistance exerted on the catheter.

Based on the above theoretical model analysis, a lower stiffness of the spring sheet results in reduced force loss transmitted to the sensor and consequently enhances detection sensitivity. However, owing to the gravitational load of the clamping structure, the spring sheet must maintain adequate stiffness in the Z direction to provide sufficient support. The challenge of achieving low stiffness in the Y direction while maintaining adequate stiffness in the Z direction in the spring sheet constitutes the focus of Section 2.3.

### 2.3. Optimization and Analysis of the Operating Force Detection Structure

In addition to adjusting the size and material properties of straight spring sheets, stiffness can be enhanced through heat treatment, surface treatment, and optimized manufacturing processes. Bent spring sheets can simultaneously improve stiffness in both the Y and Z directions for three main reasons: (1) The effective length is shortened, reducing the lever arm, which according to the principle of leverage, increases the resisting torque against deformation; (2) The load mode changes from simple tension or compression to combined bending and torsion, thereby engaging more comprehensive material properties; (3) Work hardening occurs during the bending process, resulting in increased material hardness and strength. Therefore, bent spring sheets are employed instead of straight ones to optimize structural stiffness.

**Figure 5:**
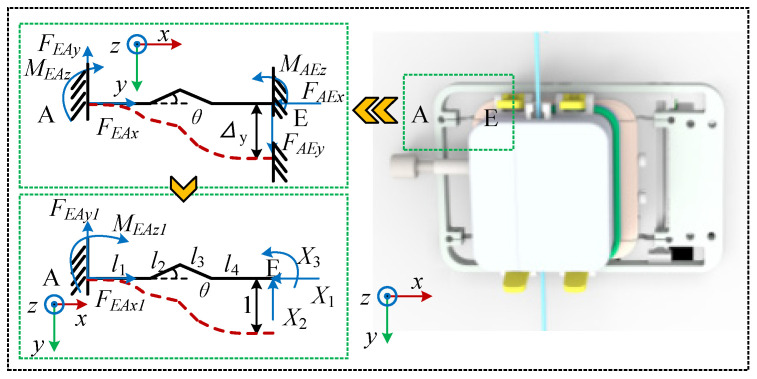
Simplified model and force analysis of a bent spring sheet.

The optimized structure is shown in Figure 5. A single bent spring sheet is analyzed by dividing its length into four sections. Their lengths are $l_{1},l_{2},l_{3},l_{4}$, the angles between $l_{2},l_{3}$ and the horizontal direction are both θ, where $l_{1}=l_{4},l_{2}=l_{3}$. Similar to the straight spring sheet analysis, one end is assumed to be fixed while a unit displacement load is applied to the opposite end. Using the fundamental equations of the force method, the constraint forces at the free end are represented by *X*_1_, *X*_2_, and *X*_3_. Force analysis is then performed to solve the free terms in the equations derived from the force method. The detailed calculation process is described in Appendix A. The results are as follows:

(8) $$\begin{array}{l} \delta_{11}=2l_{3}^{3}\sin^{2}\theta /3EI\\ \delta_{12}=\delta_{21}=(4l_{3}^{3}\sin\theta \cos\theta +3l_{3}^{2}l_{4}\sin\theta )/6EI\\ \delta_{13}=\delta_{31}=2l_{3}^{2}\sin\theta /EI\\ \delta_{22}=(12l_{3}^{2}l_{4}\cos^{2}\theta +18l_{3}l_{4}^{2}\cos\theta +8l_{3}^{3}\cos^{2}\theta )/3EI\\ +(11l_{3}^{2}l_{4}\cos\theta +6l_{3}l_{4}^{2}+8l_{4}^{3})/3EI\\ \delta_{23}=\delta_{32}=2(l_{3}l_{4}\cos\theta +l_{4}^{2}+l_{3}^{2}\cos\theta +l_{3}l_{4})/EI\\ \delta_{33}=2(l_{3}+l_{4})/EI\\ \Delta_{1c}=0\\ \Delta_{2c}=1\\ \Delta_{3c}=1/2(l_{3}\cos\theta +l_{4}) \end{array}$$

The unknown variables can be solved by substituting Equation (8) into Equation (3). Subsequently, the force equilibrium equations can determine the vertical force acting on the spring sheet.

## 3. Experimental Method and Setup

### 3.1. Performance Evaluation of Compliant Structures

**Figure 6:**
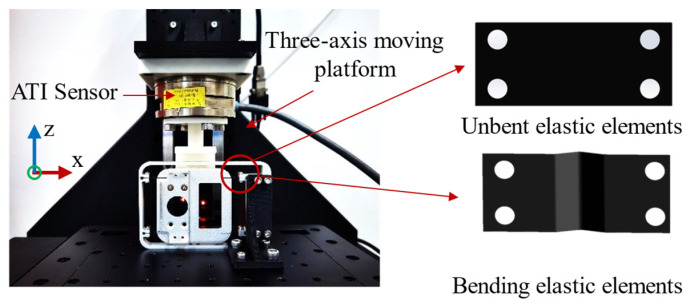
Experimental platform for spring sheet stiffness testing. **Upper right**: schematic of the straight spring sheet; **lower right**: schematic of the bent spring sheet.

To validate the spring sheet stiffness model proposed in the previous section, a dedicated test platform was constructed to compare the stiffness characteristics of straight and bent spring sheets. As shown in Figure 6, the force detection module was mounted on the base plate of a three-axis motion platform, while an ATI force sensor (SI65-5; ATI Industrial Automation, Apex, NC, USA; Z-direction force range: ±200 N; sampling frequency: 200 Hz) was installed on the *Z*-axis. The platform was lowered during the experiment until the push rod reached critical contact with the detection module. Subsequently, it was incrementally reduced by 0.05 mm within a total displacement range of 0–0.5 mm, and the output of the ATI sensor was recorded.

### 3.2. Force Detection Method Calibration Experiment

**Figure 7:**
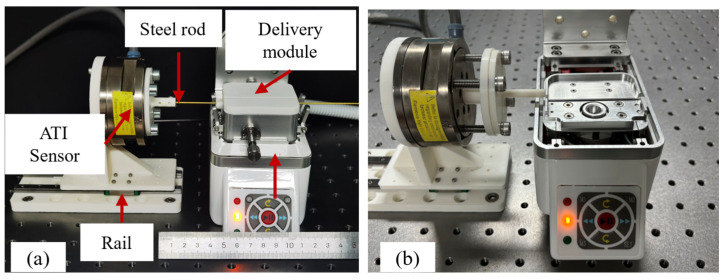
Experimental platform for the force detection module performance evaluation. (**a**) Experimental platform for evaluating the accuracy of force detection methods. (**b**) Calibration experimental platform after removing the clamping module.

A calibration platform was constructed to evaluate the force detection accuracy of the combined compliant structure and sensor, as illustrated in Figure 7a. The ATI sensor was fixed onto a linear guide rail, and a steel rod was inserted through the clamping module. A steel rod was chosen to eliminate the influence of deformation on force detection accuracy. During the experiment, the ATI sensor was repeatedly pushed in the horizontal direction, and both its *Z*-axis component and the output from the sensor within the delivery module were recorded. Each test group was repeated 10 times. Peak values from both sensors were extracted and fitted using the least squares method.

Due to the presence of components such as drive gears within the clamping module, the point of force application is not aligned with the plane of the spring sheet, resulting in torsional deformation. The theoretical model does not account for torsional deformation of the spring sheet induced by axial resistance in the steel rod. Therefore, the clamping module was removed to ensure alignment between the applied force and the spring sheet plane. The revised experimental setup is illustrated in Figure 7b. The experimental procedure was consistent with that of the calibration test. The ATI sensor was repeatedly pushed in the horizontal direction, and both its *Z*-axis output and the internal sensor readings were recorded. Each test group was repeated ten times, and peak values were extracted for least squares fitting. The adjusted force relationship is as follows:

(9) $$F_{a}=(48EI/l^{3}+k_{sensor}+k_{other})\Delta_{y}$$

### 3.3. Evaluation of the Delivery Accuracy of the Force Detection Module

**Figure 8:**
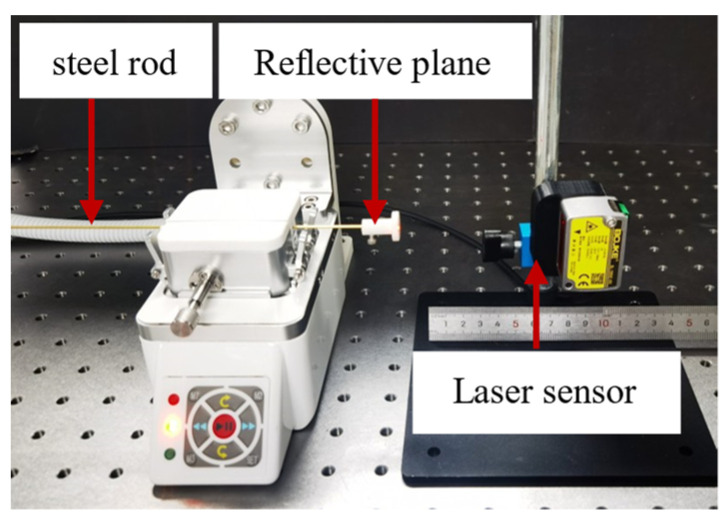
Experimental platform for evaluating the positional accuracy of the delivery module.

An axial accuracy test platform was constructed to evaluate the axial positioning accuracy of the rotary clamping and delivery module when operating instruments such as catheters or guidewires, as shown in Figure 8. A 1.6 mm diameter steel rod was used in place of the catheter to eliminate measurement errors caused by elastic deformation. The rod was mounted onto the clamping module and secured using the adjustable clamping knob. The distal end of the steel rod was attached to a reflective surface. A laser displacement sensor (model BL-100NZ-485, Shenzhen Boyi Jingke Technology Co., Ltd. Shenzhen, China) with a measurement range of ±35 mm and a repeatability of 70 μm was used. The computer controlled the rotary clamping and delivery module by sending pulses, with 81,920 pulses corresponding to one complete revolution. Pulse counts of 81,920, 163,840, 245,760, 327,680, 409,600, 491,520, 573,440, 655,360, 737,280, and 778,240 were applied to measure the resulting displacement of the steel rod. Each test condition was repeated ten times to obtain the average displacement. Theoretical displacement was calculated using the following formula:

(10) $$S=Pulse\cdot \pi \cdot d/(512\cdot i_{1}\cdot i_{2})$$

where *d* is the diameter of the rotary clamping wheel, $i_{1}$ is the reduction ratio of the harmonic reducer, and $i_{2}$ is the gear ratio of the internal gear transmission of the clamping module. We computed the mean of the deviations between the theoretical and measured values across ten independent experiments, and defined this metric as the mean detection error.

### 3.4. Comparison of Performance with ATI Sensors

**Figure 9:**
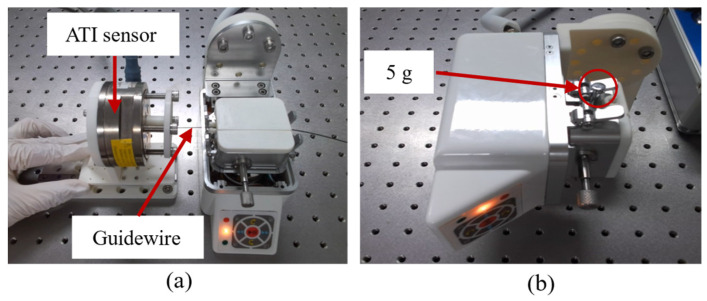
(**a**) Experimental platform for comparing the response speed of the proposed force detection method with that of the ATI sensor. (**b**) Experimental platform for evaluating the sensitivity of the force detection module.

To evaluate the response speed of the proposed method for detecting catheter or guidewire operation force, we selected a softer guidewire (diameter: 0.038 in). We established an experimental platform as shown in Figure 9a. During the experiment, the guidewire tip was initially touched manually to observe the corresponding changes in the sensor readings of the force detection module. The ATI sensor was used to apply repeated pushing and pulling forces on the guidewire tip, followed by unloading, to compare the response curves of the two sensors. The loading and unloading processes are demonstrated in Movie S1. Movie S1 illustrates the actual forces recorded by the ATI sensor and the internal sensor within the force detection module. Since the ATI sensor’s data acquisition interface has a fixed display range of (–2.5 N to 2.5 N). In contrast, the custom-designed force display interface can dynamically adjust the range based on real-time data; the difference in response is not visually prominent in the video. Therefore, the actual guidewire operation force was calculated using the previously derived formula.

### 3.5. Evaluation of the Sensitivity of the Force Detection Method

To evaluate the sensitivity of the proposed force detection method, the catheter delivery module was rotated by 90 degrees and mounted on an optical platform, as shown in Figure 9b. During the experiment, calibrated weights of 5 g, 2 g, and 1 g were applied sequentially, and the corresponding sensor responses were monitored and recorded for analysis.

### 3.6. Force Detection Module Gravity Compensation

**Figure 15:**
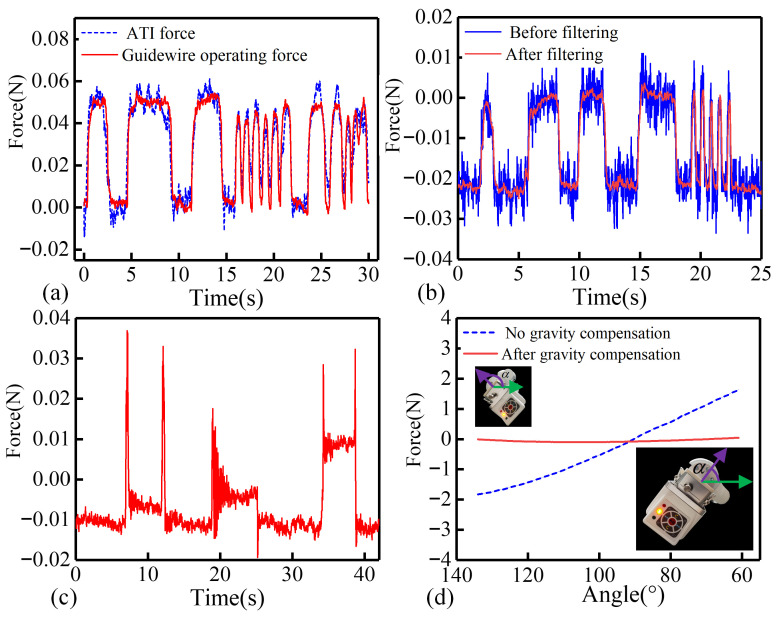
Results of response speed, sensitivity, and gravity compensation evaluation of the force detection method. (**a**) Comparison of response speed between the proposed force detection method and the ATI sensor. (**b**) Filtered sensor data using the adaptive Kalman filter. (**c**) Sensor output when 1 g, 2 g, and 5 g weights are placed on the force detection module. (**d**) Gravity compensation results of the force detection module. The green arrow indicates the horizontal direction, and the purple arrow indicates the x-axis direction of the robot arm’s end flange.

The preceding analysis examined the operating force under the condition that the catheter manipulator was installed horizontally. However, during actual catheter delivery, the catheter manipulator does not maintain a horizontal configuration; instead, the module must be tilted at a certain angle, as shown in the upper-left sub-figure of Figure 15d. The module contains motors, harmonic reducers, and clamping units. The weight of these components leads to a baseline offset in the measured force when the module is tilted. On one hand, this offset affects the force data transmitted to the master controller. The gravity-induced error in the operating force may result in inaccurate haptic feedback from the master controller. On the other hand, a zero-point offset in the force curve, even in the absence of external force, may mislead the operator. Therefore, gravity compensation for the force detection module is required, and the established compensation model is as follows:

(11) $$F_{a}=(48EI/l^{3}+k_{\mathrm{sensor}})\Delta_{y}+m\mathrm{gsin}(\alpha -90{}^{\circ})$$

where *m* is the total mass of the transmission module and the clamping module, g is the gravitational acceleration, and α is the angle between the force detection module and the horizontal plane, as illustrated in the lower-right sub-figure of Figure 15d.

In the experiment, the catheter delivery module was tilted to 135°, and the sixth joint of the robotic arm was rotated 75° clockwise to observe the resulting changes in the force curve. The experimental procedure for gravity compensation is demonstrated in Movie S3.

### 3.7. Real-Time Operating Force Detection and Verification of Vascular Phantoms

**Figure 10:**
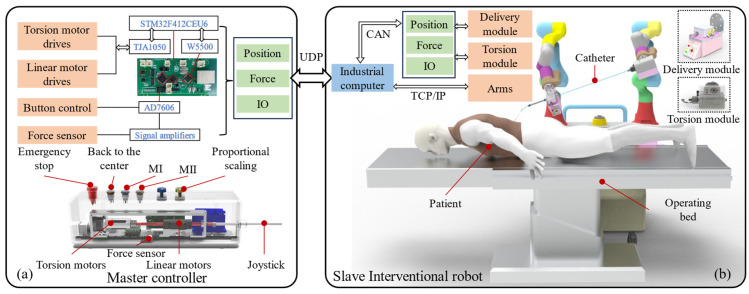
Schematic diagram of the master–slave control framework. (**a**) Structure and control framework of the master controller. (**b**) Structure and control framework of the slave interventional robot.

To evaluate the performance of our force detection module during catheter intervention in a vascular phantom and to acquire real-time operating force, remote operation of the interventional robot is achieved through a master–slave control framework. The control framework is illustrated in Figure 10. The interventional robotic system consists of two components: a master controller and a slave robotic unit. Information exchange between the master and slave units is implemented via the UDP communication protocol to ensure precise control during interventional procedures.

**Figure 11:**
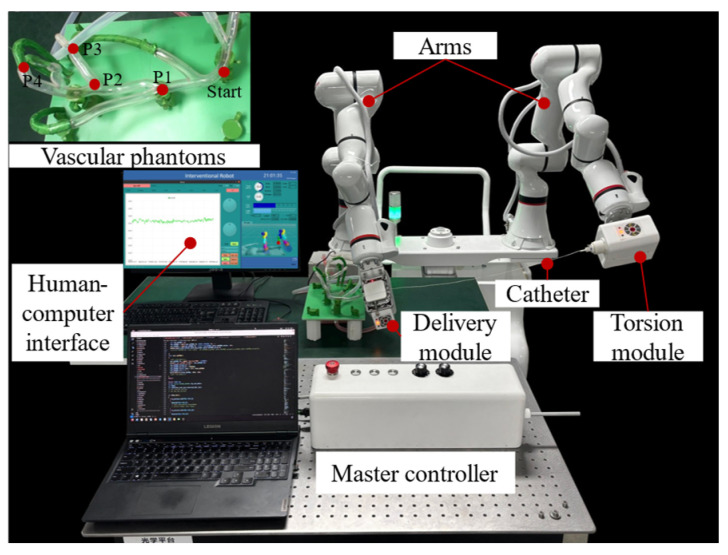
Experimental platform for catheter operation force detection in a vascular phantom.

An experimental platform was constructed for the catheter delivery task within a vascular phantom, as shown in Figure 11. The inner diameter range of the vascular model is 4–8 mm. The master controller was set to the master–slave mapping MI mode. The operator performed pushing, pulling, and retraction actions using the master joystick. After reaching the travel limit, the joystick can be automatically reset by pressing the return-to-midpoint button. Additionally, this study’s human–computer interaction interface displays the operating force curve in real time. The interface’s lower-right corner shows the robotic arm’s motion status and posture, while the upper-right corner presents motor status information. The state of the catheter inside the vascular phantom is transmitted to the interface via a camera feed. The catheter delivery process and real-time force detection are demonstrated in Movie S4.

## 4. Analysis of Experimental Results

### 4.1. Performance Evaluation Results of Compliant Structures

**Figure 12:**
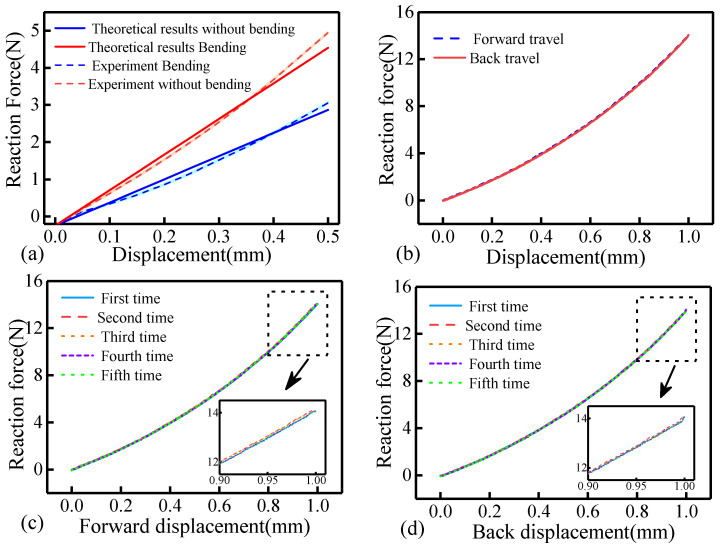
Performance evaluation of the compliant mechanism. (**a**) Force–displacement curves of the straight and bent spring sheets. Solid lines represent theoretical results, and dashed lines represent experimental measurements. (**b**) Hysteresis test results of the compliant mechanism. (**c**) Repeatability test results during loading. (**d**) Repeatability test results during unloading.

The performance evaluation results of the compliant structure are presented in Figure 12a. In contrast to the theoretical model, the experimental setup applied loading along the *Z*-axis of the platform. This choice was made because a servo motor drives the *Z*-axis, whereas stepper motors with lower positional accuracy control the X- and Y-axes. The gravitational effect of the clamping module was also taken into consideration. In Figure 12a, the gravitational force of the clamping module was considered equivalent to the initial tension measured by the ATI sensor. The sensor detected a preload tension of 0.25 N at the initial position, resulting in a downward shift in the force–displacement curve by 0.25 N. From the experimental curve, it can be observed that the stiffness remains approximately linear when the spring sheet deformation ranges from 0 to 0.4 mm, with a slope of 9.85 N/mm. The experimental results agree with the theoretical predictions, thereby validating the accuracy of the proposed model. When the deformation exceeds 0.4 mm, the stiffness curve exhibits a nonlinear trend. However, the spring sheet is directly connected to the sensor in practical applications. The sensor has a range of 2 kg, a stiffness of 66.67 N/mm, and a maximum deformation of 0.3 mm. According to relevant literature [15], the operating force of the catheter does not exceed 3 N. Therefore, the proposed stiffness model is applicable within this operational range.

The mechanism undergoes both forward and reverse strokes during operation. To evaluate the ability of the structure to return to its initial state after deformation and to assess potential hysteresis during the recovery process, the experimental setup shown in Figure 6 was used, with a loading displacement range of 0–1 mm and a loading speed of 0.1 mm/s. The results are shown in Figure 12b. It can be observed that the loading and unloading curves follow a similar trend, with no significant hysteresis observed. In addition, a repeatability test of the compliant mechanism was conducted. The loading speed remained at 0.1 mm/s, with the forward and reverse strokes set to 1 mm. The experiment was repeated five times. The results are presented in Figure 12c,d. The force–displacement responses across the five trials were highly consistent. Both the loading and unloading processes demonstrated good repeatability of the mechanism. Therefore, the mechanism satisfies the requirements for repeated loading and unloading during catheter delivery.

### 4.2. Force Detection Method Calibration Results

**Figure 13:**
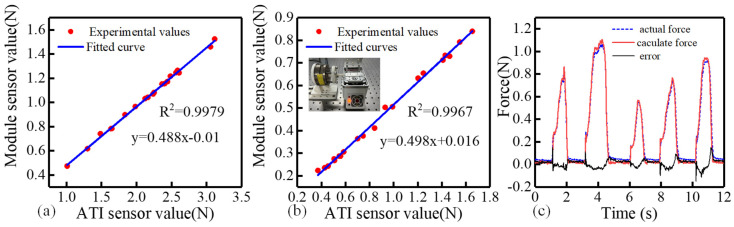
Experimental results of the force detection module performance evaluation. (**a**) Relationship between the ATI sensor data and the delivery module sensor data obtained by least squares fitting. (**b**) The relationship between the ATI sensor data and the delivery module sensor data was obtained by fitting the least squares after removing the clamping module. (**c**) Comparison between the actual force measured by the sensor and the calculated force.

The calibration results are shown in Figure 13a. A strong linear relationship was observed between the two datasets, with a correlation coefficient of 0.9979.

The calibration result after removing the clamping module, shown in Figure 13b, indicates a linear correlation coefficient of 0.9967 between the two datasets. Experimental results showed that the combined stiffness of the bent spring sheet is 9.85 N/mm, whereas the stiffness of the internal sensor is 66.67 N/mm. The theoretical model predicted that the internal sensor’s force change should be 87.1% of the ATI sensor’s output, whereas the experimental value was only 49.8%. This discrepancy does not indicate an error in the theoretical model. The discrepancy arises because the base and sensor mounting interface were assumed to be rigid in the model. However, a 3 mm thick aluminum alloy base was used in practice, which undergoes deformation under axial loading. The theoretical model was subsequently corrected based on experimental data by incorporating additional structural compliance to improve its accuracy.

Finally, the relationship between the calculated force and the actual measured force was established. The result is presented in Figure 13c. The maximum detection error is defined as the largest deviation between the theoretical and measured values, which occurs at the fifth peak in Figure 13c. The mean detection error is calculated as the sum of all deviations divided by the total number of sampling points. Based on the curve, the maximum detection error of the proposed method is 0.1482 N, and the average detection error is 0.0096 N.

### 4.3. Evaluation of the Delivery Accuracy Results of the Force Detection Module

**Figure 14:**
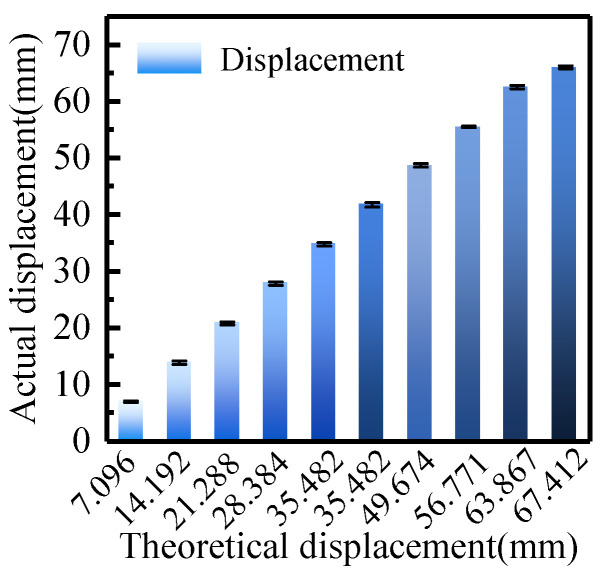
Comparison between the theoretical and actual displacements of the delivery module.

The axial accuracy results of ten experimental groups are presented in Figure 14, showing an average absolute detection error of 0.8287 mm and an average relative error of 2.19%. The primary source of error is that the diameter of the steel rod used is larger than that of a typical catheter and lacks deformability. This results in backlash between the driving and driven gears, causing desynchronization and slippage during the initial motion. Compared to the reciprocating dragging scheme, the rotary clamping scheme improves delivery efficiency at the cost of increased positioning error. However, this error level remains within clinically acceptable limits for surgical applications.

### 4.4. Comparison of Performance Results with ATI Sensors

The response speed results are presented in Figure 15a. It can be observed that the proposed method demonstrates a rapid response without noticeable delay, and its real-time performance is sufficient to meet surgical requirements. During the acquisition of force sensor data via the microcontroller, it was observed that motor-induced vibrations and environmental noise significantly affected the signal quality. An adaptive Kalman filtering algorithm was implemented to preprocess the sensor data [43]. The filtering results are illustrated in Figure 15b. The filtered signal exhibits no noticeable delay and responds rapidly to abrupt load changes. Additionally, fine features near the signal peaks are preserved.

### 4.5. Evaluation of the Sensitivity Results of the Force Detection Method

The evaluation of the Sensitivity results of the force detection method is presented in Figure 15c. Specifically, a 1 g calibrated weight was placed on the clamping module to evaluate the force response. As illustrated by the first peak in Figure 15c, the force curve shows a distinct and repeatable change, demonstrating that the proposed method can reliably resolve a 0.01 N load, demonstrating high sensitivity.

### 4.6. Force Detection Module Gravity Compensation Results

The gravity compensation experimental results are presented in Figure 15d. Without gravity compensation, as the tilt angle decreases, the force measured by the detection module shifts from negative to positive. After applying gravity compensation, the measured force remains stable as the tilt angle decreases. When the tilt angle is 90°, i.e., the catheter manipulator is in the horizontal position, the measured forces with and without gravity compensation are equal. This result validates the correctness of the proposed gravity compensation model.

### 4.7. Real-Time Operating Force Detection Results

**Figure 16:**
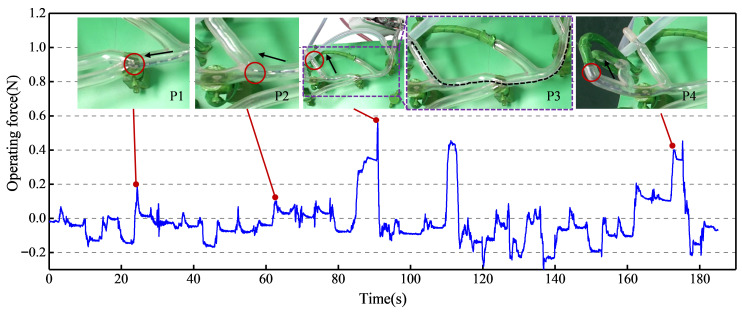
Experimental demonstration and results of catheter delivery force detection in a vascular phantom. The red circle indicates the position of the catheter tip, the black arrow indicates the direction of movement of the catheter tip, and the purple dashed line is the enlarged view.

The real-time operating force detection experimental results are presented in Figure 16. Four key points were selected for detailed analysis. P1 corresponds to the bifurcation of the first blood vessel, P2 to the bifurcation of the second vessel, P3 to the end of the second vessel branch, and P4 to the end of the third vessel branch. The catheter delivery task was accomplished using the coordinated operation of the master controller joystick and function buttons. When the catheter reached P1, it contacted the vessel wall, resulting in slight bending deformation and increased operating force to 0.19 N. Further advancement enabled entry into the lower branch of the first blood vessel. Subsequently, the master joystick was retracted, the catheter tip was oriented toward the upper branch, and delivery continued. Upon reaching P2, the catheter again contacted the vessel wall, and the force increased to 0.10 N. After fine adjustment of the tip angle, the catheter entered the upper branch of the second vessel. At P3, the end of the vessel branch was occluded. With continued advancement via the master controller, the catheter contacted the vessel wall, causing the force to first rise to 0.38 N and then peak at 0.56 N. Upon observing the force increase, the master controller was retracted, and the force immediately decreased. The catheter was then redirected toward the lower branch from P2 to continue delivery. When reaching P4, contact with the vessel wall occurred again, and the operating force increased to 0.20 N. To evaluate the sensitivity of the force detection module over extended catheter insertion distances, the master joystick was continuously pushed forward. As shown in Movie S4, the operating force increased to 0.40 N as the catheter advanced further into the vascular phantom. A slight delay in the force curve was observed due to catheter deformation, though it remained within acceptable limits for surgical requirements. After the final retraction, the force returned to its initial baseline.

## 5. Discussion

To better demonstrate the advantages of the proposed operating force detection method, relevant studies on catheter force detection were reviewed, and their results were compared. As shown in Table 1, the comparison metrics included maximum detection error, maximum relative error, average detection error, and resolution.

A comparison of the results indicates that although the maximum detection error of the proposed method is slightly higher than that reported by Yu et al. [21]. The average detection error is reduced by 63.5%. Compared with other studies, the maximum and average detection errors are lower. However, existing studies have not reported the resolution of force detection. This metric was evaluated in our research, and the results demonstrate that the resolution of the proposed method reaches 0.01 N, indicating high sensitivity. High-resolution force detection enables surgeons to perceive the contact state between surgical instruments and tissues more accurately, allowing them to identify potential risks promptly and make timely adjustments. Moreover, it provides surgeons with more realistic and refined force feedback. When operating surgical robots remotely, surgeons experience a heightened sense of immersion, as if manipulating the instruments directly with their hands.

Through its unique structural design, our operation module demonstrates high-precision manipulation and high-resolution force detection capabilities. Compared with existing solutions, this design structurally eliminates the influence of friction on force detection accuracy, thereby significantly enhancing the precision and stability of interventional force measurement. High-resolution force detection serves as a prerequisite for achieving highly realistic haptic feedback. Furthermore, it provides an effective solution for implementing force feedback in the master controller. However, regarding the deformation compensation of the fixed plate mentioned earlier, we will select suitable high-strength materials (such as high-hardness aluminum alloy or stainless steel) to manufacture the base in future studies, and design sets of bases with different structural strengths. By conducting comparative calibration experiments on these optimized bases, we will quantitatively analyze the correlation between the base’s structural strength, the amount of deformation, and the required compensation, thereby verifying the rationality of the current compensation strategy and further optimizing its calculation model.

## 6. Conclusions

This paper proposes a method for detecting the operational force of an interventional robot catheter by integrating a compliant mechanism with a sensor. A stiffness model of the compliant mechanism was developed to optimize the force detection structure, and its accuracy was validated experimentally. Based on this, we used an adaptive Kalman filter algorithm to filter the force signal and completed the gravity compensation of the force detection module. Finally, we completed the real-time force detection verification of catheter delivery in a vascular phantom. Experimental results demonstrate that the proposed method achieves high resolution, enables precise detection of small surgical forces, and maintains low detection error. The designed master–slave interventional robotic system supports real-time force detection and visual feedback, contributing to surgical safety.

## Figures and Tables

**Table 1:** Comparison with existing research parameters.

Reference	Passive Grasping Success Rate	Maximum Relative Detection Error	Average Detection Error (N)	Resolution (N)
Wang et al. [22]	Not mentioned	Not mentioned	0.048	Not mentioned
Zhao et al. [14]	0.151	4.55%	0.0345	Not mentioned
Yu et al. [21]	0.098	8.23%	0.0263	Not mentioned
Ren et al. [23]	0.1925	21.9%	0.023	Not mentioned
This paper	0.1482	13.4%	0.0096	0.01

**Figure A1:**
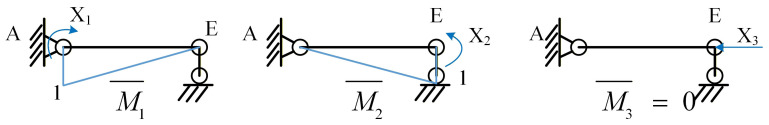
Using straight spring sheet analysis, bending moment diagrams when X1, X2, and X3 act individually.

**Figure A2:**
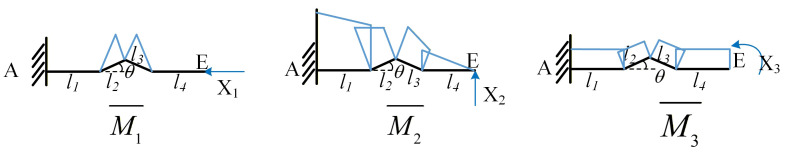
Using bending spring sheet analysis, bending moment diagrams when X1, X2, and X3 act individually.

## Data Availability

The raw data supporting the conclusions of this article will be made available by the authors upon request.

## Supplementary Materials

The following supporting information can be downloaded at: https://www.mdpi.com/article/10.3390/biomimetics11040229/s1, Movie S1: Comparison of Performance with ATI Sensors; Movie S2: Evaluation of the Sensitivity of the Force Detection Method; Movie S3: Force Detection Module Gravity Compensation; Movie S4: Real-Time Operating Force Detection and Verification of Vascular Phantoms.

## Appendix A

Using straight leaf spring analysis, the bending moment diagrams for *X*_1_, *X*_2_, and *X*_3_ acting individually are as shown in Figure A1:

The coefficients in the equation can be determined using graphical multiplication.

$$\begin{array}{l} \delta_{11}=\sum \int \bar{M_{1}}\times \bar{M_{1}}/EIds=\frac{1}{EI}\cdot \frac{1}{2}\cdot l\cdot 1\cdot \frac{2}{3}=l/3EI\\ \delta_{12}=\sum \int \bar{M_{1}}\times \bar{M_{2}}/EIds=\frac{1}{EI}\cdot \frac{1}{2}\cdot l\cdot 1\cdot \frac{1}{3}=l/6EI\\ \delta_{22}=\sum \int \bar{M_{2}}\times \bar{M_{2}}ds=\frac{1}{EI}\cdot \frac{1}{2}\cdot l\cdot 1\cdot \frac{2}{3}=l/3EI\\ \delta_{13}=\delta_{23}=\delta_{33}=0\\ \Delta_{1c}=-\Delta_{2c}=1/l\\ \Delta_{3c}=0 \end{array}$$

The bending moment diagrams for *X*_1_, *X*_2_, and *X*_3_ acting individually are shown in Figure A2, based on the analysis of the selected bending spring sheet:

The coefficients in the equation can be determined using graphical multiplication.

$$\delta_{11}=\sum \int \bar{M_{1}}\times \bar{M_{1}}/EIds=2\cdot \frac{1}{EI}\cdot \frac{1}{2}\cdot l_{3}\sin\theta \cdot l_{3}\cdot \frac{2}{3}l_{3}\sin\theta$$

$$\begin{array}{l} \delta_{12}=\sum \int \bar{M_{1}}\times \bar{M_{2}}/EIds=\frac{1}{EI}[\frac{l_{3}}{6}(2\cdot 0\cdot (l_{3}\cos\theta +l_{4})+2l_{3}\sin\theta (l_{3}\cos\theta +l_{4})+2(l_{3}\cos\theta +l_{4})\cdot l_{3}\sin\theta )]\\ =\frac{1}{EI}[\frac{l_{3}}{6}(4l_{3}^{2}\sin\theta \cos\theta +3l_{3}l_{4}\sin\theta )]\\ =\frac{1}{6EI}[4l_{3}^{3}\sin\theta \cos\theta +3l_{3}^{2}l_{4}\sin\theta ] \end{array}$$

$$\delta_{13}=\sum \int \bar{M_{1}}\times \bar{M_{3}}/EIds=2\cdot \frac{1}{EI}\cdot \frac{1}{2}\cdot l_{2}\sin\theta \cdot l_{3}\cdot 1=2l_{3}^{2}\sin\theta /EI$$

$$\delta_{21}=\delta_{12}$$

$$\begin{array}{l} \delta_{22}=\sum \int \bar{M_{1}}\times \bar{M_{2}}/EIds=\frac{l_{4}}{6EI}[2\cdot 4{\left(l_{3}\cos\theta +l_{4}\right)}^{2}+2{\left(2l_{3}\cos\theta +l_{4}\right)}^{2}+2\cdot 2(l_{3}\cos\theta +l_{4})\cdot (2l_{3}\cos\theta +l_{4})]\\ +\frac{l_{3}}{6EI}[2{\left(2l_{3}\cos\theta +l_{4}\right)}^{2}+2{\left(l_{3}\cos\theta +l_{4}\right)}^{2}+2(2l_{3}\cos\theta +l_{4})(l_{3}\cos\theta +l_{4})]\\ +\frac{l_{3}}{6EI}[2{\left(l_{3}\cos\theta +l_{4}\right)}^{2}+2l_{4}^{2}+2(l_{3}\cos\theta +l_{4})\cdot l_{4}]+\frac{1}{EI}[\frac{1}{2}\cdot l_{4}\cdot l_{4}\cdot \frac{2}{3}\cdot l_{4}]\\ =\frac{l_{4}}{6EI}[24l_{3}^{2}\cos^{2}\theta +36l_{3}l_{4}\cos\theta +14l_{4}^{2}]+\frac{l_{3}}{6EI}[14l_{3}^{2}\cos^{2}\theta +16l_{3}l_{4}\cos\theta +6l_{4}^{2}]\\ +\frac{l_{3}}{6EI}[2l_{3}^{2}\cos^{2}\theta +6l_{3}l_{4}\cos\theta +6l_{4}^{2}]+\frac{l_{4}^{3}}{6EI}\\ =(12l_{3}^{2}l_{4}\cos^{2}\theta +18l_{3}l_{4}^{2}\cos\theta +8l_{3}^{3}\cos^{2}\theta )/3EI+(11l_{3}^{2}l_{4}\cos\theta +6l_{3}l_{4}^{2}+8l_{4}^{3})/3EI \end{array}$$

$$\begin{array}{l} \delta_{23}=\sum \int \bar{M_{2}}\times \bar{M_{3}}ds=\frac{l_{4}}{6EI}[2\cdot 2(l_{3}\cos\theta +l_{4})+2(2l_{3}\cos\theta +l_{4})+(l_{2}l_{3}\cos\theta +l_{4})+2(l_{3}\cos\theta +l_{4})]\\ +\frac{l_{3}}{6EI}[2(2l_{3}\cos\theta +l_{4})+2(l_{3}\cos\theta +l_{4})+l_{3}\cos\theta +l_{4}+2l_{3}\cos\theta +l_{4}]\\ +\frac{l_{3}}{6EI}[2(l_{3}\cos\theta +l_{4})+2l_{4}+l_{4}+l_{3}\cos\theta +l_{4}]+\frac{1}{EI}[\frac{1}{2}\cdot l_{4}\cdot l_{4}\cdot 1]\\ =\frac{2}{EI}[l_{3}l_{4}\cos\theta +l_{4}^{2}+{l_{3}}^{2}\cos\theta +l_{3}l_{4}] \end{array}$$

$$\delta_{32}=\delta_{23}$$

$$\delta_{33}=\sum \int \bar{M_{2}}\times \bar{M_{2}}ds=2\frac{1}{EI}(1\cdot l_{4}\cdot 1+1\cdot l_{3}\cdot 1)=\frac{2}{EI}(l_{3}+l_{4})$$

## References

[1] Li N., Wang Y., Zhao H., Ding H. (2024). Robotic Systems Design in Endovascular Treatment. IEEE Trans. Med. Robot. Bionics.

[2] Ren B., Zhao Y., Zhang J., Li H., Li K., Zhang J. (2023). The Critical Technologies of Vascular Interventional Robotic Catheterization: A Review. IEEE Sens. J..

[3] Bao X., Guo S., Yang C., Zheng L. (2024). Haptic Interface With Force and Torque Feedback for Robot-Assisted Endovascular Catheterization. IEEE/ASME Trans. Mechatron..

[4] Feng K., Xu Q., Wong S.F., Zi B. (2025). Design and Development of a Teleoperated Telepresence Robot System With High-Fidelity Haptic Feedback Assistance. IEEE Trans. Autom. Sci. Eng..

[5] Jin X., Guo S., Song A., Shi P., Li X., Kawanishi M. (2024). A Novel Robotic Platform for Endovascular Surgery: Human–Robot Interaction Studies. IEEE Trans. Instrum. Meas..

[6] Li H., Zhou X.H., Xie X.L., Liu S.Q., Feng Z.Q., Hou Z.G. (2024). CASOG: Conservative Actor–Critic With SmOoth Gradient for Skill Learning in Robot-Assisted Intervention. IEEE Trans. Ind. Electron..

[7] Kanagaratnam P., Koa-Wing M., Wallace D.T., Goldenberg A.S., Peters N.S., Davies D.W. (2008). Experience of robotic catheter ablation in humans using a novel remotely steerable catheter sheath. J. Interv. Card. Electrophysiol..

[8] Bismuth J., Duran C., Stankovic M., Gersak B., Lumsden A.B. (2013). A first-in-man study of the role of flexible robotics in overcoming navigation challenges in the iliofemoral arteries. J. Vasc. Surg..

[9] Pappone F.C.C. (2009). Stereotaxis Niobe^®^ magnetic navigation system for endocardial catheter ablation and gastrointestinal capsule endoscopy. Expert Rev. Med. Devices.

[10] Pereira V.M., Cancelliere N.M., Nicholson P., Radovanovic I., Drake K.E., Sungur J.-M., Krings T., Turk A. (2020). First-in-human, robotic-assisted neuroendovascular intervention. J. Neurointerv. Surg..

[11] Venkiteswaran V.K., Sikorski J., Misra S. (2019). Shape and contact force estimation of continuum manipulators using pseudo rigid body models. Mech. Mach. Theory.

[12] Wang L., Guo C., Zhao X. (2022). Magnetic soft continuum robots with contact forces. Extrem. Mech. Lett..

[13] Abdelaziz M.E.M.K., Zhao J., Gil Rosa B., Lee H.-T., Simon D., Vyas K., Li B., Koguna H., Li Y., Demircali A.A. (2024). Fiberbots: Robotic fibers for high- precision minimally invasive surgery. Sci. Adv..

[14] Zhao Y., Guo S., Xiao N., Wang Y., Li Y., Jiang Y. (2018). Operating force information on-line acquisition of a novel slave manipulator for vascular interventional surgery. Biomed. Microdevices.

[15] Bao X., Guo S., Guo Y., Yang C., Shi L., Li Y., Jiang Y. (2022). Multilevel Operation Strategy of a Vascular Interventional Robot System for Surgical Safety in Teleoperation. IEEE Trans. Robot..

[16] Cao S., Guo S., Guo J., Wang J., Zhang Y., Zhang Y., Yang P., Liu J. (2024). A Reciprocating Delivery Device-Based Endovascular Intervention Robot With Multimanipulators Collaboration. IEEE Trans. Instrum. Meas..

[17] Yan Y., Guo S., Lin Z., Lyu C., Zhao D., Yang P., Zhang Y., Zhang Y., Liu J. (2023). Grasping-Force-Based Passive Safety Method for a Vascular Interventional Surgery Robot System. IEEE Trans. Instrum. Meas..

[18] Lyu C., Guo S., Yan Y., Zhang Y., Zhang Y., Yang P., Liu J. (2024). Deep-Learning-Based Force Sensing Method for a Flexible Endovascular Surgery Robot. IEEE Trans. Instrum. Meas..

[19] Song H.-S., Yi B.-J., Won J.Y., Woo J. (2022). Learning-based catheter and guidewire-driven autonomous vascular intervention robotic system for reduced repulsive force. J. Comput. Des. Eng..

[20] Shi C., Ishihara H. (2023). Performance Evaluation of a Vascular Interventional Surgery Robotic System with Visual-Based Force Feedback. Machines.

[21] Yu H., Wang H., Chang J., Liu W., Wang F., Niu J. (2023). Design and evaluation of vascular interventional robot system for complex coronary artery lesions. Med. Biol. Eng. Comput..

[22] Wang S., Shen H., Liu Z., Xie L. (2024). TCN-Based Distal Force Feedback Strategy of a Vascular Interventional Surgery Robot. IEEE Sens. J..

[23] Ren B., Zhao Y., Zhang J., Wang C., Li H., Zhang J. (2025). Multi-point bending operation force detection method of catheter-based on rotary clamping delivery. Sens. Actuators A Phys..

[24] Ren B., Zhao Y., Zhang J., Zhang J. (2024). Catheter Friction Drive Based on Rotary Clamp Delivery. J. Mech. Eng..

[25] Zhang R., Dong L., Wang X., Tian M., Su H. (2025). A Sensor-Less Guider Contact Force Estimation Approach for Endovascular Slender Robot-Assisted Guidance System. IEEE Trans. Instrum. Meas..

[26] Nguyen C.C., Thai M.T., Hoang T.T., Davies J., Phan P.T., Zhu K., Wu L., Brodie M.A., Tsai D., Ha Q.P. (2023). Development of a soft robotic catheter for vascular intervention surgery. Sens. Actuators A Phys..

[27] Dagnino G., Kundrat D., Kwok T.M.Y., Abdelaziz M., Chi W., Nguyen A., Riga C., Yang G.Z. (2023). In-Vivo Validation of a Novel Robotic Platform for Endovascular Intervention. IEEE Trans. Biomed. Eng..

[28] Kim S., Bae S., Lee W., Jang G. (2024). Magnetic Navigation System Composed of Dual Permanent Magnets for Accurate Position and Posture Control of a Capsule Endoscope. IEEE Trans. Ind. Electron..

[29] Yang Z., Yang L., Zhang M., Xia N., Zhang L. (2023). Ultrasound-Guided Wired Magnetic Microrobot with Active Steering and Ejectable Tip. IEEE Trans. Ind. Electron..

[30] Fu S., Chen B., Li D., Han J., Xu S., Wang S., Huang C., Qiu M., Cheng S., Wu X. (2023). A Magnetically Controlled Guidewire Robot System with Steering and Propulsion Capabilities for Vascular Interventional Surgery. Adv. Intell. Syst..

[31] Li Z., Li J., Wu Z., Chen Y., Yeerbulati M., Xu Q. (2024). Design and Hierarchical Control of a Homocentric Variable-Stiffness Magnetic Catheter for Multiarm Robotic Ultrasound-Assisted Coronary Intervention. IEEE Trans. Robot..

[32] Gunduz S., Albadawi H., Oklu R. (2020). Robotic Devices for Minimally Invasive Endovascular Interventions: A New Dawn for Interventional Radiology. Adv. Intell. Syst..

[33] Sa J., Park J., Jung E., Kim N., Lee D., Bae S., Lee Y., Jang G. (2023). Separable and Recombinable Magnetic Robot for Robotic Endovascular Intervention. IEEE Robot. Autom. Lett..

[34] Li Z., Xu Q. (2024). Multi-Section Magnetic Soft Robot with Multirobot Navigation System for Vasculature Intervention. Cyborg Bionic Syst..

[35] Mehdi S.Z., Janssens W., Gielen M., Vanderschueren E., Ourak M., Verslype C., Laleman W., Poorten E.V. FBG-based Actuation and Data Driven Contact Detection for Smart Steerable Instruments. Proceedings of the 2025 IEEE/RSJ International Conference on Intelligent Robots and Systems (IROS).

[36] Ben Hassen R., Lemmers A., Delchambre A. (2023). Tri-Axial Force Sensor in a Soft Catheter Using Fiber Bragg Gratings for Endoscopic Submucosal Dissection. IEEE Sens. J..

[37] Arefinia E., Jagadeesan J., Patel R.V. (2024). Machine-Learning-Based Multi-Modal Force Estimation for Steerable Ablation Catheters. IEEE Trans. Med. Robot. Bionics.

[38] Lyu Z., Xu Q. (2023). Design and testing of a large-workspace XY compliant manipulator based on triple-stage parallelogram flexure. Mech. Mach. Theory.

[39] Lyu Z., Cao Y., Wang M.Y., Xu Q. (2024). Concept design of a monolithic compliant series-elastic actuator with integrated position and two-level force sensing. Mech. Mach. Theory.

[40] Lyu Z., Xu Q. (2024). A compliant constant-force mechanism with sub-Newton force and millimeter stroke output. Sens. Actuators A Phys..

[41] Xu Q. (2012). New Flexure Parallel-Kinematic Micropositioning System With Large Workspace. IEEE Trans. Robot..

[42] Gao X., Deng J., Wang W., Chang Q., Sun J., Liu J., Zhang S., Liu Y. (2024). A Compact 2-DOF Cross-Scale Piezoelectric Robotic Manipulator With Adjustable Force for Biological Delicate Puncture. IEEE Trans. Robot..

[43] Qi W., Qin W., Yun Z. (2024). Closed-loop state of charge estimation of Li-ion batteries based on deep learning and robust adaptive Kalman filter. Energy.

